# Real-World Efficacy and Safety of Gemcitabine, Cisplatin, Plus Immune Checkpoint Inhibitors in Biliary Tract Cancer: A Retrospective Analysis

**DOI:** 10.3390/cancers18162555

**Published:** 2026-08-09

**Authors:** Mai Kitahara, Kei Saito, Yoko Oki, Noriyuki Kuniyoshi, Shuzo Nomura, Mariko Fujisawa, Hirofumi Kogure

**Affiliations:** Division of Gastroenterology and Hepatology, Department of Internal Medicine, Nihon University School of Medicine, 30-1 Oyaguchi-Kamicho, Itabashi-ku, Tokyo 173-8610, Japan; kitahara.mai@nihon-u.ac.jp (M.K.);

**Keywords:** biliary tract cancer, durvalumab, elderly patients, pembrolizumab

## Abstract

**Background**: Based on the demonstrated survival benefits of gemcitabine plus cisplatin (GemCis) therapy in the TOPAZ-1 and KEYNOTE-966 trials, GemCis plus immune checkpoint inhibitor (ICI) therapy has become a standard first-line treatment for unresectable biliary tract cancer. However, its safety and efficacy in elderly patients and those requiring biliary drainage remain insufficiently evaluated in real-world practice. We aimed to evaluate the efficacy and safety of GemCis plus ICI therapy, with a focus on elderly patients and those requiring biliary drainage. **Methods:** We retrospectively analyzed the clinical characteristics of patients with unresectable biliary tract cancer treated with GemCis plus ICI at our institution between April 2022 and September 2025. Progression-free survival (PFS) was analyzed using the Kaplan–Meier method, and prognostic factors were examined using Cox proportional hazards models. PFS and immune-related adverse events (irAEs) were compared between durvalumab and pembrolizumab. **Results:** Of the 51 patients diagnosed with unresectable biliary tract cancer during the observation period, 26 (51.0%) received GemCis plus ICI therapy. The median age was 73 years (range, 46–83 years), and 15 (57.7%) patients were male. Distant metastases were present in 22 patients (84.6%), and biliary drainage was performed in 15 (57.7%). The primary tumor sites were intrahepatic cholangiocarcinoma (*n* = 8), perihilar cholangiocarcinoma (*n* = 6), gallbladder cancer (*n* = 11), and cancer of unknown primary origin (*n* = 1). The ICIs used were durvalumab in 18 patients and pembrolizumab in eight patients. The median follow-up period was 214 days (range, 30–1114). The response rate was 28.6%, the disease control rate was 90.5%, and the transition rate to ICI maintenance therapy was 29.2%. IrAEs occurred in six patients (23.1%), with a significantly higher frequency in the pembrolizumab group than in the durvalumab group (50.0% vs. 11.1%). The median PFS was 8.2 months in the durvalumab group and 8.9 months in the pembrolizumab group, with no significant difference (*p* = 0.88). Multivariate analysis incorporating age, performance status, metastatic burden, and liver function confirmed that age ≥ 75 years remained the only independent predictor of shorter PFS (HR 5.62; 95% CI, 1.35–23.4; *p* = 0.02), whereas ICI regimen and biliary drainage were not significant prognostic factors. **Conclusions:** In clinical practice, GemCis plus ICI therapy showed no statistically significant difference in efficacy between ICI regimens in this small cohort, although this comparison was not statistically powered. Given the exploratory nature of these findings, the lower incidence of immune-related adverse events (irAEs) observed in the durvalumab group should be interpreted with caution. Age ≥ 75 years was associated with shorter PFS. Careful patient selection is warranted when considering GemCis plus ICI therapy in elderly patients with biliary tract cancer.

## Simple Summary

In recent years, the combination of standard chemotherapy (gemcitabine plus cisplatin) with immune checkpoint inhibitors (ICIs) has become the first-line treatment for biliary tract cancer. However, the performance of this combination in elderly patients and those requiring biliary drainage in routine clinical practice is not well understood. In this study, we retrospectively analyzed the clinical data of 26 patients treated at our hospital and found that combination therapy was effective overall, with a median progression-free survival of approximately eight months. Importantly, patients aged ≥ 75 years had significantly shorter survival than younger patients, suggesting the need for individualized patient selection in the elderly. In contrast, the requirement for biliary drainage did not significantly affect treatment outcomes, indicating that ICI-based therapy may be feasible under appropriate management. Additionally, immune-related side effects occurred more frequently with pembrolizumab than with durvalumab, which may help guide the choice of ICI agent in clinical practice.

## 1. Introduction

Biliary tract cancer is a malignant tumor with a poor prognosis and a 5-year survival rate of approximately 20% [1]. With an aging population, the number of patients is increasing [2]. The standard treatment for unresectable advanced biliary tract cancer is chemotherapy, with gemcitabine plus cisplatin (GemCis) therapy being the gold standard [3,4]. In 2022, Ioka et al. demonstrated the superiority of gemcitabine, cisplatin, and S-1 (GCS) therapy over GemCis [5]. Consequently, GCS therapy has become one of the standard treatments in Japan. Okamoto et al. reported that, in elderly patients with unresectable biliary tract cancer aged 75 years or older, the rates of treatment discontinuation due to intolerance and transition to second-line chemotherapy were comparable to those in non-elderly patients, and age was not an independent predictor of overall survival [6]. Similarly, Yamada et al., in a subgroup analysis of the JCOG1113 trial, demonstrated that the clinical outcomes of combination chemotherapy in elderly patients were similar to those observed in younger patients [7]. In addition, prior biliary drainage has been reported to have no significant impact on the efficacy or tolerability of GemCis-based chemotherapy in patients with biliary tract cancer [8]. Moreover, GemCis plus durvalumab therapy has been shown to be safely administered in a real-world cohort that included patients with biliary drainage [9]. Furthermore, the efficacy of immune checkpoint inhibitors (ICIs) has been reported in other malignancies. Clinical trials in 2022 demonstrated the superiority of GemCis plus durvalumab [10] and GemCis plus pembrolizumab over GemCis [11]. Since becoming standard treatments, immunotherapies such as GemCis plus durvalumab and GemCis plus pembrolizumab have been widely adopted.

While immunotherapy has raised expectations for improved long-term prognosis [12], studies evaluating its safety and efficacy in elderly patients and in those requiring biliary drainage in biliary tract cancer remain limited, representing an unmet medical need. Therefore, we conducted a retrospective analysis of patients with biliary tract cancer treated with immunotherapy to evaluate its safety and efficacy in elderly patients and those requiring biliary drainage.

## 2. Methods

This study was a retrospective, single-center, exploratory cohort study.

### 2.1. Patients and Procedures

Patients aged ≥ 18 years who were treated at our institution between April 2022 and September 2025 with GemCis plus durvalumab or GemCis plus pembrolizumab for locally advanced or metastatic biliary tract cancer were included in this retrospective study.

The inclusion criteria were as follows: (1) pathological diagnosis of unresectable biliary tract cancer, and (2) age of 18 years or older. The exclusion criteria were as follows: (1) Eastern Cooperative Oncology Group performance status (PS) of 3 or higher; (2) cases in which the attending physician determined that combination chemotherapy was not appropriate; and (3) prior administration of combination chemotherapy with another regimen.

The drug administration schedule and dosage were as follows: 1. The GemCis plus durvalumab group (Group D) received durvalumab (1500 mg) on day 1 of each cycle in combination with gemcitabine (1000 mg/m^2^) and cisplatin (25 mg/m^2^), which were administered on days 1 and 8 of each cycle. 2. The GemCis plus pembrolizumab group (Group P) received pembrolizumab (200 mg) on day 1 of each cycle in combination with gemcitabine (1000 mg/m^2^) and cisplatin (25 mg/m^2^) on days 1 and 8 of each cycle. Both regimens were administered in 3-week cycles.

In Group D, durvalumab monotherapy (1500 mg) was administered every four weeks after the completion of GemCis. In Group P, gemcitabine on days 1 and 8 of each cycle plus pembrolizumab on day 1 of each cycle were administered every three weeks after completion of cisplatin. Treatment in both groups continued until disease progression or the occurrence of severe adverse events (AEs).

This study was approved by the Institutional Review Board of Nihon University School of Medicine (approval number: RK-260414-4) and conducted in accordance with the Declaration of Helsinki. Written informed consent was waived due to the retrospective nature of the study.

### 2.2. Outcome

The primary endpoint was the identification of factors associated with PFS. Secondary endpoints included antitumor response and the incidence of irAEs. Prognostic factor analyses for PFS were conducted, and AEs were graded according to the National Cancer Institute Common Terminology Criteria for Adverse Events, Version 5.0.

### 2.3. Statistical Analysis

The chi-squared or Fisher’s exact test was used to compare categorical variables. Student’s *t*-test or the Mann–Whitney U-test was used to compare continuous variables. PFS was estimated using the Kaplan–Meier method and analyzed with the log-rank test. Univariate and multivariate Cox proportional hazards regression analyses were performed to identify prognostic factors for PFS. All reported *p* values were from two-sided tests; *p* values < 0.05 were considered statistically significant. All statistical analyses were performed using JMP 19 software (SAS Institute, Cary, NC, USA).

## 3. Results

### 3.1. Patient Characteristics

**Figure 1:**
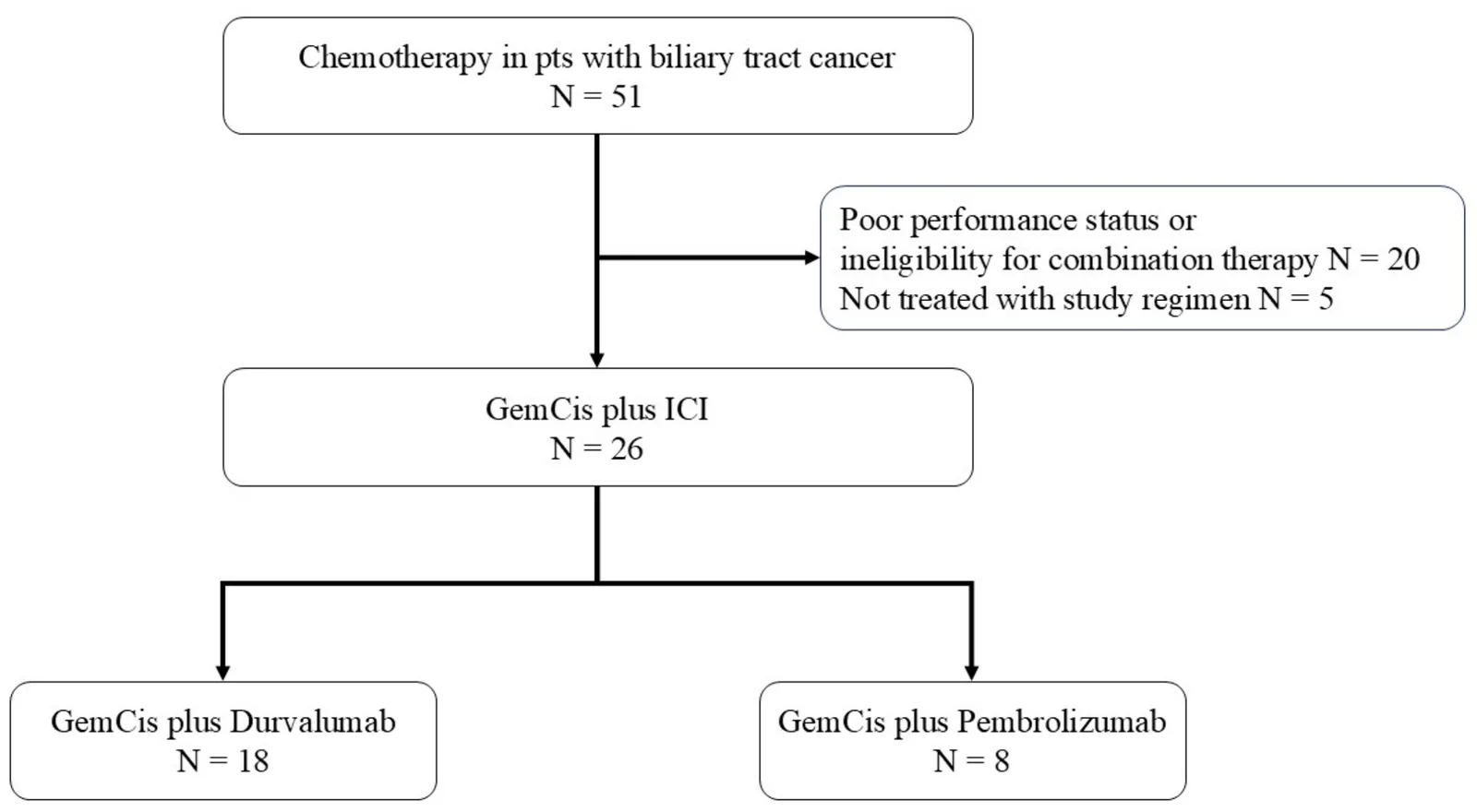
Flow chart of this study.

Of the 51 patients diagnosed with unresectable biliary tract cancer during the observation period, 26 (51.0%) received GemCis combined with immune checkpoint inhibitors (GemCis plus ICI) (Figure 1). The 25 patients were excluded due to poor performance status or ineligibility for combination therapy (*n* = 20) and not being treated with the study regimen (*n* = 5). The median age was 73 years (range, 46–83), and 15 patients were male (57.7%). Regarding the Eastern Cooperative Oncology Group performance status (PS), four patients had PS 0, 21 had PS 1, and one had PS 2. Distant metastases were observed in 22 patients (84.6%) (Table 1).

The primary tumor sites included intrahepatic cholangiocarcinoma (*n* = 8), perihilar cholangiocarcinoma (*n* = 6), gallbladder cancer (*n* = 11), and cancer of unknown primary origin (*n* = 1). Biliary drainage was performed in 15 (57.7%) patients. The median serum CA19-9 level was 341.6 U/mL (range, 1.2–1,177,007).

The modified Glasgow Prognostic Score was 0 in eight patients, 1 in ten, and 2 in eight. The modified albumin–bilirubin (ALBI) grade was 1 in eight patients, 2a in six patients, 2b in eleven patients, and 3 in one patient.

Regarding concomitant oral medications, 12 patients (46.2%) received antibiotics, 9 (34.6%) angiotensin II receptor blockers, 6 (23.1%) statins, 5 (19.2%) proton pump inhibitors, 3 (11.5%) non-steroidal anti-inflammatory drugs, 2 (7.7%) metformin hydrochloride, 2 (7.7%) sodium–glucose cotransporter 2 inhibitors, and 1 (3.8%) received probiotics (Table 1).

### 3.2. ICI Therapy

The ICIs administered were durvalumab in 18 patients and pembrolizumab in eight. Seven patients (29.2%) transitioned to maintenance therapy.

Among patients who received maintenance therapy, two were aged ≥75 years and three were male (42.8%). All patients had a PS of 1, and distant metastases were present in all patients (100%). The primary tumor sites included gallbladder cancer in three patients (42.8%), intrahepatic cholangiocarcinoma in two (28.6%), perihilar cholangiocarcinoma in one (14.3%), and cancer of unknown primary origin in one (14.3%). Biliary drainage was required in three patients (42.8%) (Table 2).

### 3.3. Antitumor Efficacy and Adverse Events

**Figure 2:**
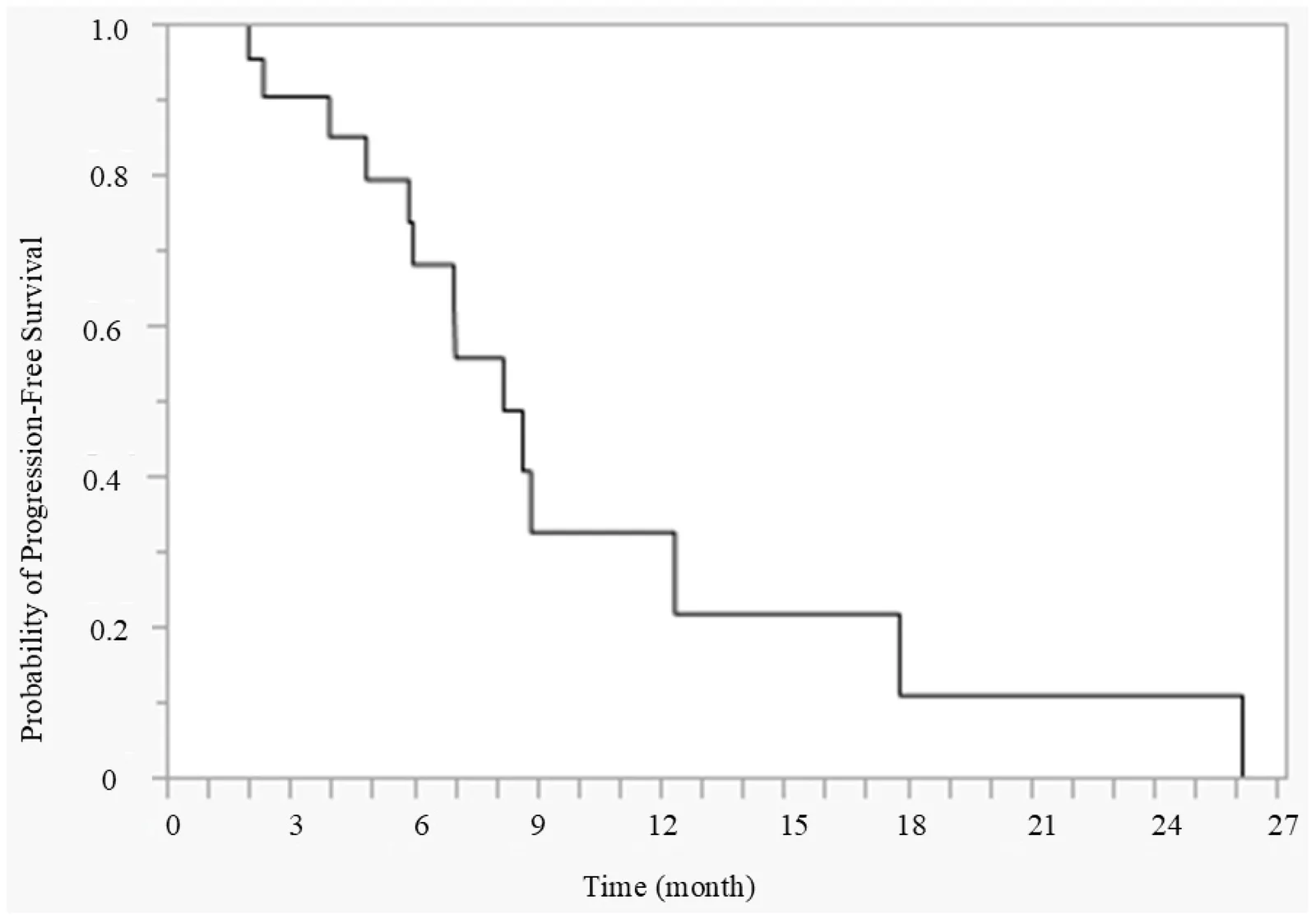
Kaplan–Meier curve for progression-free survival (PFS).

The median follow-up period was 214 days (range, 30–1114). The overall response rate was 28.6%, and the disease control rate was 90.5% (Table 3). The median PFS of the entire cohort treated with GemCis plus ICI was 8.2 months (95% confidence interval [CI], 5.9–12.4) (Figure 2).

**Figure 3:**
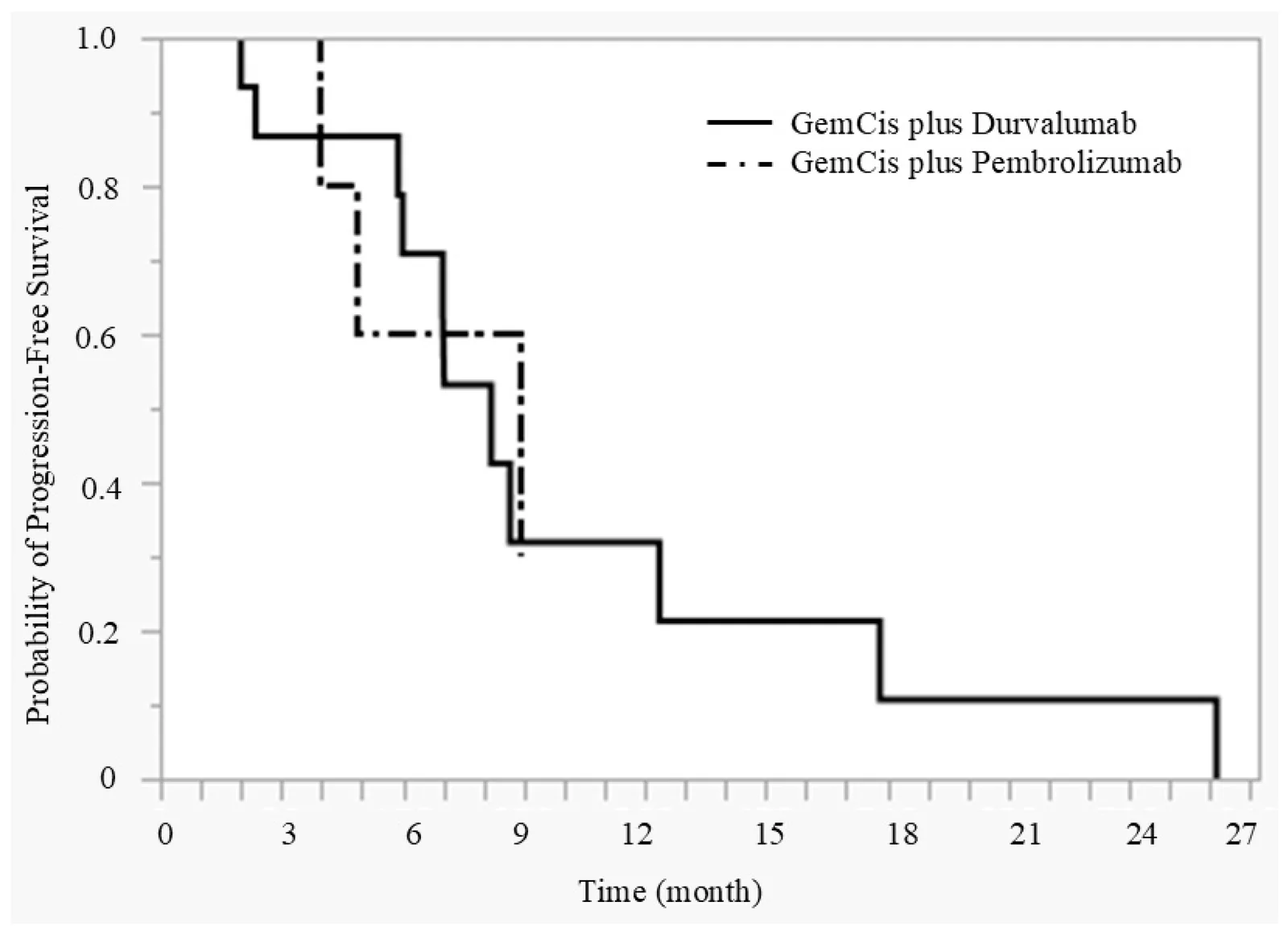
Kaplan–Meier curves for progression-free survival (PFS) in the GemCis plus durvalumab and GemCis plus pembrolizumab groups.

When analyzed by ICI type, the median PFS was 8.2 months (95% CI, 5.9–12.4) in Group D and 8.9 months (95% CI, 4.0–not estimable) in Group P, with no significant difference between the groups (*p* = 0.88) (Figure 3).

Grade ≥ 3 hematologic toxicities occurred in 12 patients, and no severe non-hematologic adverse events were observed. IrAEs occurred in six patients (23.1%), including interstitial pneumonia in two (33.3%), thyroid dysfunction in two (33.3%), rash in one (16.7%), and nephritis in one (16.7%). No severe irAEs were observed (Table 3).

**Figure 4:**
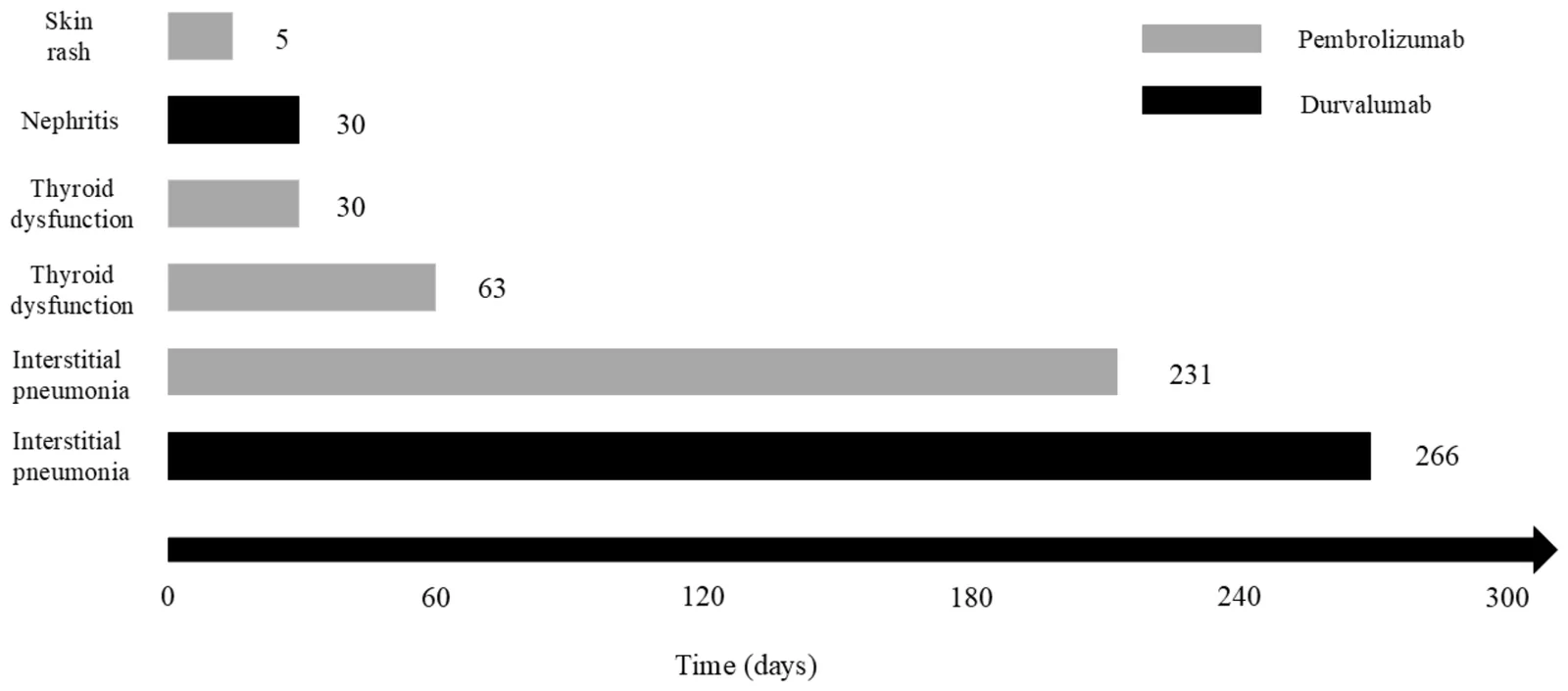
Time to onset of immune-related adverse events (irAEs) in patients treated with pembrolizumab and durvalumab.

Figure 4 shows the types of irAEs and the timing of their onset. Rash and thyroid dysfunction occurred early, whereas interstitial pneumonia occurred later.

The incidence of irAEs was 50.0% in Group P and 11.1% in Group D during the observation period.

The median PFS was 8.2 months (95% CI, 5.9–12.4) in the GemCis plus ICI group.

The median PFS was 8.2 months (95% CI, 5.9–12.4) in the GemCis plus durvalumab group and 8.9 months (95% CI, 4.0–not estimable) in the GemCis plus pembrolizumab group. No significant difference was observed between the two groups (*p* = 0.88, log-rank test).

The time from treatment initiation to the onset of each irAE is shown for pembrolizumab (gray bars) and durvalumab (black bars). The numbers indicate the number of days until onset.

### 3.4. Prognostic Factor Analysis for PFS

In the univariate Cox proportional hazards regression analysis, age ≥ 75 years was significantly associated with shorter PFS (hazard ratio, 4.55; 95% CI, 1.26–18.3; *p* = 0.02). Multivariate analysis incorporating age, performance status, metastatic burden, and liver function confirmed that age ≥ 75 years remained the only independent predictor of shorter PFS (HR 5.62; 95% CI, 1.35–23.4; *p* = 0.02) (Table 4).

## 4. Discussion

Our retrospective study found that GemCis plus ICI was administered to 34.6% of the study population aged ≥ 75 years; however, age ≥ 75 years was significantly associated with shorter PFS, indicating that appropriate patient selection is required.

The TOPAZ-1 and KEYNOTE-966 trials demonstrated the safety and efficacy of GemCis plus ICI in biliary tract cancer [9,10]. However, they either excluded elderly patients or included only a small number of such patients. In real-world clinical settings, elderly patients with biliary tract cancer are common, necessitating the collection of practical data. Our retrospective study included 34.6% of patients aged ≥ 75 years, representing a cohort aligned with clinical practice. The decision to administer GemCis plus ICI to elderly patients is at the discretion of the treating physician. The demonstrated safety suggests that patient selection may have been appropriate.

Organ function and geriatric functional assessments are considered useful indicators [13,14,15,16]. Our study was retrospective, and although these assessments were limited, we collected data on liver and kidney function, and only patients with preserved organ function were included in the study. The finding that advanced age was associated with poorer PFS suggests that even among patients eligible for GemCis plus ICI, careful consideration is required when selecting this regimen for older individuals, in light of prior evidence indicating the prognostic impact of age and comorbidity in advanced biliary tract cancer [17].

Several systematic reviews and meta-analyses have reported that antibiotic exposure may adversely affect the therapeutic efficacy of ICI treatment [18,19]. Biliary drainage is frequently required, and stent-related cholangitis during ICI treatment is a clinically relevant concern. In our cohort, more than half of the patients underwent biliary drainage; however, the presence of biliary drainage was not significantly associated with PFS. These findings suggest that ICI therapy can be administered to patients with biliary tract cancer requiring biliary drainage under appropriate clinical management. Similarly, a history of antibiotic use prior to ICI initiation was evaluated as a potential contributing factor, given that antibiotic exposure is likely more common in patients undergoing biliary drainage. However, it was not identified as a statistically significant predictor of PFS in our analysis.

In addition to antibiotics, probiotics and proton pump inhibitors affect the therapeutic efficacy of ICIs [20,21], whereas metformin, angiotensin II receptor blockers, and statins affect the efficacy of chemotherapy [22,23,24]. This study examined the medication history of patients. The results showed that no oral medication affected treatment efficacy. Given the small sample size and limited number of patients taking these medications, this remains a topic for future research.

IrAEs are adverse events that require careful monitoring during ICI therapy. In our study, irAEs occurred in 23.1% of patients, and no grade 3 or higher irAEs were observed, a frequency comparable to that reported previously. A notable finding was the difference in the frequency of irAEs between durvalumab and pembrolizumab. The difference in irAE frequency may be related to the distinct mechanisms of action of durvalumab, a PD-L1 inhibitor, and pembrolizumab, a PD-1 inhibitor [25,26]. While some reports suggest that a higher irAE frequency correlates with treatment efficacy [27,28], our findings suggest that durvalumab and pembrolizumab may provide comparable PFS outcomes in patients with biliary tract cancer. However, this comparison was not statistically powered, and the finding should therefore be considered exploratory rather than confirmatory. Although randomized controlled trials are needed to compare safety and efficacy, these results provide useful information for considering which agent to use.

Our study had some limitations. First, the sample size was small, and the study design was retrospective. Notably, only 9 patients were elderly. Although age was identified as a prognostic factor for shorter PFS, the small sample size limits the statistical reliability of this finding; therefore, it should be interpreted with caution. The numbers are insufficient for a direct comparison of durvalumab and pembrolizumab, and there is a potential bias in the selection of patients who received GemCis plus ICI. Second, the retrospective nature of the study limited the variables examined. Assessments of organ function and comprehensive geriatric assessment in the elderly population were not systematically performed. Future prospective studies with larger cohorts are needed to validate these findings. Third, this study was not designed as a comparative efficacy trial between GemCis plus durvalumab and GemCis plus pembrolizumab; therefore, direct comparisons of efficacy outcomes between the two groups should be interpreted with caution. In addition, maintenance therapy differed between Group D and Group P, which may have influenced both toxicity and efficacy outcomes.

## 5. Conclusions

In real-world practice, GemCis plus ICI therapy appeared to be effective in patients with biliary tract cancer, and irAEs were generally manageable. In multivariate analysis, age ≥ 75 years was independently associated with shorter PFS, suggesting that appropriate patient selection may be warranted when considering GemCis plus ICI in elderly patients with biliary tract cancer. Biliary drainage status did not appear to notably influence PFS, suggesting that ICI therapy may be administered with appropriate management in patients requiring biliary drainage. These findings are based on a small, single-center retrospective cohort without formal statistical power calculations and should therefore be regarded as hypothesis-generating trends rather than confirmatory evidence. Validation in larger prospective studies is warranted.

## Figures and Tables

**Table 1:** Baseline Characteristics of the Study Patients.

	*n* = 26
Age	73 (46–83)
≥75	9 (34.6%)
Sex, male	15 (57.7%)
PS, 0/1/2	4/21/1
Stage, Locally/Metastatic	4/22
Localization	
Gallbladder	11 (42.3%)
Intrahepatic bile duct	8 (30.8%)
Perihilar bile duct	6 (23.1%)
Cancers of Unknown Primary Origin	1 (3.8%)
Biliary drainage	15 (57.7%)
CA19-9, U/mL	341.6 (1.2–1,177,007)
NLR	4.45 (0.7–11.2)
PLR	165.5 (72.3–344.2)
mGPS, 0/1/2	8/10/8
mALBI grade, 1/2a/2b/3	8/6/11/1
Durvalumab/Pembrolizumab	18/8
Maintenance Therapy Implementation Cases	7 (29.2%)
Use of Medications	
Antibiotics	12 (46.2%)
ARB	9 (34.6%)
Statin	6 (23.1%)
PPI	5 (19.2%)
Aspirin	4 (15.4%)
NSAIDs	3 (11.5%)
Metformin Hydrochloride	2 (7.7%)
SGLT2i	2 (7.7%)
Probiotics	1 (3.8%)

ARB, Angiotensin II Receptor Blocker; mALBI, modified Albumin–Bilirubin grade; mGPS, modified Glasgow Prognostic Score; NLR, Neutrophil-to-Lymphocyte Ratio; NSAIDs, Non-Steroidal Anti-Inflammatory Drugs; PLR, Platelet-to-Lymphocyte Ratio; PPI, Proton Pump Inhibitors; PS, Performance Status; SGLT2i, Sodium–glucose cotransporter 2 inhibitor.

**Table 2:** Maintenance Therapy Implementation Cases.

	*n* = 7
75 years old and older	2 (28.6%)
Sex, male	3 (42.8%)
PS, 1	7 (100%)
Metastatic	7 (100%)
Localization	
Gallbladder	3 (42.8%)
Intrahepatic bile duct	2 (28.6%)
Perihilar bile duct	1 (14.3%)
Cancer of unknown primary origin	1 (14.3%)
Biliary drainage	3 (42.8%)

PS, Performance Status.

**Table 3:** Tumor Response and Adverse Events.

	*n* = 26
Observation period, days	214 (30–1114)
Complete Response	0%
Partial Response	28.6%
Stable Disease	61.9%
Progressive Disease	9.5%
Response rate	28.6%
Disease control rate	90.5%
Hematologic toxicity	
Neutropenia, Grade1-4	22 (84.6%)
Neutropenia, Grade3-4	5 (19.2%)
Anemia, Grade1-4	26 (100%)
Anemia, Grade3-4	6 (23.1%)
Thrombocytopenia, Grade1-4	21 (80.8%)
Thrombocytopenia, Grade3-4	1 (3.8%)
irAE	6 (23.1%)
Interstitial pneumonia	2 (33.3%)
Thyroid dysfunction	2 (33.3%)
Rash	1 (16.7%)
Nephritis	1 (16.7%)

irAE, Immune-related Adverse Events.

**Table 4:** Risk factors of progression-free survival.

		Univariate		Multivariate	
Factors		HR (95%CI)	*p* Value	HR (95%CI)	*p* Value
Age	≥75	4.55 (1.26–18.3)	0.02	5.62 (1.35–23.4)	0.02
Sex	Male	1.28 (0.42–4.04)	0.66		
PS	0 (vs. 1 or 2)	1.90 (0.39–7.42)	0.39	2.97 (0.53–16.7)	0.22
Stage	Metastatic	1.00 (0.26–6.60)	1.00	2.07 (0.29–15.0)	0.47
Primary	Gallbladder	2.73 (0.81–10.6)	0.11		
Biliary drainage	Yes	2.31 (0.74–8.60)	0.15		
DM	Yes	0.54 (0.15–1.69)	0.30		
CA19-9	≥100	1.48 (0.47–5.01)	0.50		
NLR	≥3.0	1.74 (0.52–6.17)	0.37		
PLR	≥200	2.14 (0.62–6.86)	0.22		
mGPS	0 (vs. 1 or 2)	1.00 (0.30–3.32)	1.00		
mALBI score	1 (vs. 2a, 2b, or 3)	1.02 (0.31–3.06)	0.98	0.96 (0.28–3.31)	0.96
ICI	Durvalumab	1.11 (0.32–5.12)	0.87		
Antibiotics	Yes	1.45 (0.45–4.48)	0.52		
ACE/ARB	Yes	2.11 (0.61–6.80)	0.23		
Statin	Yes	1.74 (0.40–6.17)	0.40		
PPI	Yes	1.75 (0.26–7.06)	0.51		
Aspirin	Yes	2.18 (0.54–7.47)	0.26		
NSAIDs	Yes	0.32 (0.02–1.69)	0.21		

ACE, Angiotensin-Converting Enzyme; ARB, Angiotensin II Receptor Blocker; DM, Diabetes Mellitus; ICI, Immune Checkpoint Inhibitor; mALBI, modified Albumin–Bilirubin grade; mGPS, modified Glasgow Prognostic Score; NLR, Neutrophil-to-Lymphocyte Ratio; NSAIDs, Non-Steroidal Anti-Inflammatory Drugs; PLR, Platelet-to-Lymphocyte Ratio; PPI, Proton Pump Inhibitors; PS, Performance Status.

## Data Availability

The data that support the findings of this study are not publicly available due to ethical restrictions and patient privacy concerns. The data are available from the corresponding author upon reasonable request, subject to institutional review board approval.

## Abbreviation

ACE, angiotensin-converting enzyme; AEs, adverse events; ALBI, albumin–bilirubin grade; ARB, angiotensin II receptor blocker; CA19-9, carbohydrate antigen 19-9; CGA, comprehensive geriatric assessment; CI, confidence interval; DM, diabetes mellitus; GCS, gemcitabine plus cisplatin plus S-1; GEM, gemcitabine; GemCis, gemcitabine plus cisplatin; HR, hazard ratio; ICI, immune checkpoint inhibitor; irAEs, immune-related adverse events; mALBI, modified albumin–bilirubin grade; mGPS, modified Glasgow prognostic score; NLR, neutrophil-to-lymphocyte ratio; NSAIDs, non-steroidal anti-inflammatory drugs; PD-1, programmed cell death protein 1; PD-L1, programmed death-ligand 1; PFS, progression-free survival; PLR, platelet-to-lymphocyte ratio; PPI, proton pump inhibitors; PS, performance status; SGLT2i, sodium–glucose cotransporter 2 inhibitor.
